# 

**Published:** 1893-12

**Authors:** 


### INDEX TO VOLUME XIV.

A.

A Brief Review, 15.
A Contribution to the Study of Erosion, 241.
A Contribution to the Study of the Development of the Enamel, 881.
A Few Thoughts on the Combination of Filling-Materials, 570.
A New Apparatus for maintaining Anæsthesia without a Face-Piece, 757.
A Plea for more Scientific Professional Methods, 21.
A Simple and Efficient Strengthener for Lower Vulcanite Dentures, 876.
Abbott, Frank, D.D.S., on Bichloride of Mercury and Chloride of Zinc, 561.
  on Teeth of the Lower Jaw at Birth, 721.
Abscess of the Maxillary Sinus, Treatment of, 800.
Acidum Chlorociticum, 182.
Affections of the Teeth, 641.
Agathin, a New Anti-Neuralgic, 240.
Allport, Dr. W. W., Obituary, 391.
  Resolutions of Respect to, 469, 629.
Alumni New York College of Dentistry, 235.
Amalgam as a Filling-Material, 572.
  To make Joints Tight with, 582.
American Academy of Dental Science, 118, 433, 519, 602, 662, 801.
  Dental Association, 34, 109, 269, 362, 477, 635, 715.
  Dental Degrees all rejected in Great Britain, 549.
  Diplomas in England, 624.
American Electro-Therapeutic Association, 234.
Among the Ancient Hawaiians, 735.
An Abnormal Position of a Left Central, 653.
An Act to regulate Dentistry in Wyoming, 720.
An Alleged Death from Encalyptus Oil, 880.
An Explanation, 548.
An Interesting Case, 813.
Anæsthesia, and the Use of a New Ether Inhaler, 401.
  A New Apparatus for maintaining, 757.
  Cocaine-Injections for the Production of, 769.
  Notes on the History of, 900.
Anatomy, Manual for Students—Review of, 222.
Andrews, R. R., A.M., D.D.S., on Prehistoric Crania from South America, 914.
  The Development of the Enamel, 881.
Angle, Dr. Edward H., System of Regulation and Retention of the Teeth, 223.
Antiseptic Dentistry, 869.
Argenti Nitras (Silver Nitrate), 153, 175, 238, 398, 707.
Aristol, 175.
  Iodoform *versus*, 877.
Arizona, An Act to regulate the Practice of Dentistry in, 717.
  Meeting of the Board of Examiners of, 717.
Arsenical Applications in Case of Congested Pulps, 857.
Asaprol, 240.
Assistant Editor, 218.
Association, American Dental, 34, 109, 173, 269, 362, 477, 635, 715.
  California State Dental, 480, 716.
  Central Dental, of Northern New Jersey, 66, 209, 535, 558.
  Colorado State Dental, 623.
  Missouri State Dental, 480, 874.
  Northern Ohio, 236.
  South Carolina State Dental, 560, 636.
  Southern Dental, 639.
  Woman's Dental, of the United States, 875, 926.
Associations at Chicago, Special Notice of, 639.

B.

Bacteria in Caries, 879.
Beale, Dr. Alonzo P., Obituary, 146.
Berger, John, D.D.S., on Diaphtherin in Dental Practice, 584.
Bibliography: A Compend of Dental Pathology and Dental Medicine. By Geo. W. Warren, D.D.S. (Second Edition), 549.
  A Practical Treatise on Artificial Crown- and Bridge-Work. By George Evans, D.D.S. (Third Edition), 390.
  A Series of Questions and Answers for Dental Students. By F. J. S. Gorgas, D.D.S., 76.

Bibliography:
A Study of the Degeneracy of the Jaws of the Human Race. By Eugene S. Talbot, M.D., D.D.S., 219.
All Around the Year 1893, 78.
Anatomy: Manual for Students. Fred. J. Brockway, M.D., 222.
Books of the Month, 78.
Catching's Compendium of Practical Dentistry for 1892. By B. H. Catching, D.D.S., Atlanta, Ga., 306.
Columbian Edition. Letters from a Mother to a Mother. By "Mrs. M. W. J." (Third Edition), 628.
Die Mikroorganismen der Mundhöhle. Von W. D. Miller, Dr. Méd. et Phil., 75.
Formulaire pratique pour les Maladies de la Bouche et des Dents. Par G. Viau, Professeur à l'École Dentaire de Paris, 628.
Histology, Pathology, and Bacteriology, 146.
Lippincott's Magazine, 77.
Materia Medica and Therapeutics. By L. F. Warner, M.D., 146.
Methods of Filling Teeth. By Rodrigues Ottolengui, M.D.S., 141.
Note-Book for Dental Students. By James F. Rymer, 222.
Orthodontia. By S. H. Guilford, D.D.S. (Second Edition), 389.
Physiology, A Manual of. By Frederick A. Manning, M.D., 223.
R. L. Polk & Co.'s Dental Register of the United States, 863.
Richardson's Mechanical Dentistry (Sixth Edition), Revised and Edited by Geo. W. Warren, D.D.S., 934.
The Angle System of Regulation and Retention of the Teeth. By Edward H. Angle, D.D.S., 223.
The Rise, Fall, and Revival of Dental Prosthesis. By B. J. Cigrand, B.S., D.D.S., 221.
Bichloride of Mercury, 176.
and Chloride of Zinc in the Treatment of Pulpless Teeth and Alveolar Abscesses, 561.
Experiments with, 753.
Bishop, Dr. J. Adams. Experiences in treating Cleft Palates, 406.
on Injuries and Diseases of the Mouth, and their Treatment, 321.
Black, G. V., D.D.S., on Report of Committee on Dental Nomenclature, 837.
Blakeman, Dr. R. I., on Filling Roots with Gutta-Percha dissolved in Chloroform, 267.
Bleichsteiner, Dr. Anthony, on Cocaine-Injections for the Production of Anæsthesia, 769.
Board of Examiners of the Territory of Arizona, 717.
Bödecker, C. F. W., D.D.S. The Doctrine of Inflammation, 1.
Bogue, E. A. Correction, 813.
Bonwill, W. G. A., on New Method of Clasped Plates *versus* Movable or Unmovable Bridge-Work, 86.
Books of the Month, 78.
Boracine (Tetraborate of Soda), 798.
Bridge-Work, Clasped Plates *versus*, 87.
Dr. Richmond's Method, 81, 161, 481.
Briggs, Charles P., M.D., D.M.S., on Cocaine, 817.
Briggs, Edward C., M.D., D.M.S. The Dentist as a Prescriber of Drugs, 567.
Brockway, Fred. J., M.D. Compend of Anatomy, 222.
Brown, Geo. V. I., on Prudence and Gutta-Percha in Crown- and Bridge-Work, 782.
Brown, Dr. Peter. The Disadvantages of High-Tension Circuits of Dental Work, etc., 483.
Business and Professional Methods, 814.

C.

California State Dental Association, 48, 716.
Caracatsanis, D., M.D., on Method of inducing Local Anæsthesia by Cocaine, 775.
Case, Dr. C. S., on Some Principles governing the Development of Facial Contours in the Practice of Orthodontia, 776.
Case of Fracture and Suppuration around the Incisive Alveolar Portion of the Superior Maxillary Bone, and Reimplantation in the same of a Lateral Incisor Eight Days after the Injury, 842.
Cassidy, J. C., D.D.S., M.D. Elements of Chemistry and Dental Materia Medica, 468.
Causes of Failure in Dental Operations, 25.
Caush, Dr. D. E., on Some Changes that take place in and around the Pulp-Canal, 794.
Cell Metamorphosis, 659.
Census Law, Discussion on, 362.
Central Dental Association of New Jersey, 66, 209, 535, 558.
Central Incisor, the Pedigree of, 833.
Change in Time, American Dental Association, 477.
Changes that take place in and around the Pulp-Canal, 794.
Character as demanded in Professional Life, 493.
Chicago Dental Club, 452.
Chicago Dental Society, 815.
Resolutions on the Death of Dr. W. W. Allport, 629.
Chloride of Zinc and Bichloride of Mercury, 561.
Cigrand, B. J., B.S., D.D.S. Rise, Fall, and Revival of Dental Prosthesis, 221.
Clasped Plates *versus* Bridge-Work, 87.
Clasps, the Use of, 353.
Cleft Palate, Experiences in treating, 406.
Coca-Base, A New, as a Local Anæsthetic, 237.
Cocaine, 817.
Injections for the Production of Anæsthesia, 769.

Cocaine, Method of inducing Local Anæsthesia by, 775.
Colorado State Dental Association, 623.
Columbian Dental Club, 559.
Dental Club of Chicago, 549.
Dental Congress, The Importance of attending, 384.
Special Notice, 635.
Common Law, Some of the Rights and Duties of Dentists at, 586.
Congenital Defects of the Enamel, 759.
Congress Programme—Important Correction, 388.
Connecticut Valley Dental Society, 719.
Resolutions of, 720.
Corrections of Deformities of the Oral Region, 416.
Correspondence: Association of Northern New Jersey, 149.
Correction, 227, 813.
Effect of Imagination, 936.
Further Use of Nitrate of Silver, 707.
Living in Chicago during the Fair, 629.
New Mode of using Nitrate of Silver, 153.
Our Duty in regard to the World's Columbian Dental Congress, 471.
Philadelphia County Dental Society, 151.
Professional Ethics, 705.
Reply to Dr. Page, 704.
to Dr. Taft, 868.
The Dental Protective Association of the United States, 473.
The Eleventh International Medical Congress. Section of Odontology, 153.
The Pan-American Medical Congress, 226.
Crania of Native Hawaiians, 871.
Prehistoric, from Central America, 914.
Cravens, Dr., on Matrices, 55.
Crouse, Dr. J. N. Living in Chicago during the Fair, 629.
The Dental Protective Association, 473.
Crowns, Genese, 413.
Crown- and Bridge-Work. By Dr. George Evans. Review of, 390.
Prudence and Gutta-Percha in, 782.
By Dr. C. M. Richmond, 81, 161, 481.
Crystal Mat Gold, Dr. Kirk on, 97.
Current News: Announcement of the First Pan-American Medical Congress, 229, 232.
Annual Meeting of the International Dental Publishing Company, 228.
California State Dental Association, 480.
Change in Time, American Dental Association, 477.
Chicago Dental Society, 815.
College Commencements, 820.
Columbian Dental Club of Chicago, 559.
Current News:
Committee on Exhibits, World's Columbian Dental Congress—Important Notice, 479.
Dental Law in the Territory of New Mexico, 815.
Harvard Odontological Society—Annual Meeting, 317.
Illinois State Dental Society and Iowa State Dental Society—Joint Meeting, 399.
Memorial of the British Dental Association to the General Medical Council of Great Britain, 633.
Missouri State Dental Association, 480, 874.
National Association of Dental Examiners, 712.
National Association of Dental Faculties, 707.
North Carolina State Dental Society, 319.
Officers of the Philadelphia County Dental Society, 159.
Pennsylvania State Dental Examining Board, 480.
Recent Patents, 79, 159, 477, 637, 816, 874, 940.
San Francisco Dental Association, 159.
Southern Dental Association, 815.
Taft, Chas. H., Removal of, 159.
The Eleventh International Congress of Medicine, 313.
The University of Pennsylvania, 939.
The World's Columbian Dental Congress—Special Notice, 478.
World's Columbian Dental Congress, 154, 229, 553.
Curtis G. Lenox, M.D., D.D.S. The Value of Post-Graduate Instruction, 486.

### D.

Darby, Edwin T., D.D.S., Correction, 227.
Davenport, Dr. W. S., on Twisted Wire for Regulating Teeth, 247.
Deformities of the Oral Region, Corrections of, 416.
Degeneracy of the Jaws of the Human Race, Review of, 219.
Denis, Dr., on Boracine (Tetraborate of Soda) 798.
Dental Colleges, Number of, 45.
Diagnosis, 889.
Education, Legislation, and Degrees, 48.
Examiners, National Board of, 636.
Law in the Territory of New Mexico, 815.
Nomenclature, Report of the Committee on, 837.
Pathology and Dental Medicine, A Compend of, 628.
Practice, Diaphtherin in, 584.
Prosthesis, Rise, Fall, and Revival of, Review of, 221.

Dental Protective Association, 467, 473.
Register of the United States, R. L. Polk & Co.'s, 865.
Development of the Enamel, A Contribution to the Study of, 881.
Diagnosis, Dental, 889.
Diagram of the Structure of Connective Tissue, 6, 7.
Diaptherin in Dental Practice, 584.
Die Mikroorganismen der Mundhöhle, Review of, 75.
Diplomas in England, American, 624.
Disadvantage of High-Tension Circuits for Dental Work, 483.
Discussion on A Brief Study of the Molar Teeth of the Proboscidiæ, 34.
Acidum Chlorociticum (Trichloracetic Acid), 183.
Anæsthesia and the New Ether Inhaler, 433.
Argenti Nitras, 183.
Aristol, 183.
Arsenical Applications in Case of Congested Pulps, 857.
Banding Fractured Roots, 118.
Causes of Failure in Dental Operations, 66.
Collar-Crowns as a Cause of Pericemental Inflammation, 622.
Corrective Dentistry: Its Present Status, 529.
Crown- and Bridge-Work, 519.
Crownless Teeth, 199.
Crystal Mat Gold, 127.
Dental Diagnosis, 927.
Doctrine of Inflammation, 31.
Does the Quality of Amalgam depend on Atomic Formulæ? 676.
Erosion, 288.
Ethyl Chloride, 183.
Europhen, 183.
Excluding Exhibits from Society Meetings, 367.
Hydrogen Peroxide Solutions, 672.
Hyperostosis, 852.
Hypnotism, 446.
Investigations relative to Prehistoric Crania, 279.
Iron, Syrup of Chloride of, 183.
Lime formations in the Pulp-Chamber, 917.
Matrices, 109.
New Preparations of Plastic Filling-Material, 860.
Obtunding Sensitive Dentine, 664.
Orthodontia: Its Present Status. What are the Simplest and most Universally Applied Forms of Apparatus, and most Efficient Retaining Fixtures? 844.
Power of Examining Boards, 60.
Root-Filling, 118, 285.
Should Examining Boards have Power to grant Certificates of Qualification to Undergraduates? 801.
The Census Law, 362.
Discussion on The Effect produced upon the Tissues of the Oral Cavity by the Internal Administration of Certain Drugs, 273.
The Usé of Cocaine in extracting Pulps, 436.
The Value of Post-Graduate Instruction, 535.
Transplantation, 109.
Treatment of Nearly Exposed Pulps, 510.
Treatment of Putrescent Pulps, 209.
What are the Advantages and Disadvantages of the Use of the Matrix? 801.
Does the Quality of Amalgam depend on Atomic Formulæ? 676.
Doctrine of Inflammation, 1.
Douche Tooth-Brush, 234.
Dr. Allport Dead, 305.
Drilling Cavities in Artificial Teeth, 938.
Drugs, The Dentist as a Prescriber of, 567.
Duties of the Hour, 701.

E.

Eddy, Forrest G., M.D., on Character as demanded in Professional Life, 493.
Edentulous Jaws of the Human Subject, The Forms of, 872.
Editorial: All American Dental Degrees rejected in Great Britain, 549.
American Diplomas in England, 624.
An Explanation, 548.
Assistant Editor, 218.
Columbian Dental Club of Chicago, 549.
Dr. Allport Dead, 305.
Dr. Crouse, on Expenses to the Congress, 627.
Dr. Palmer, on Manual Training, 139.
Duties of the Hour, 701.
Forces which make for Progress, 73.
Let us reason together, 71.
New Dental School in New York, 141.
New Weekly Journal, 140.
Nomenclature as applied to Dentistry, 863.
Our Recent Dead, 386.
Pan-American Medical Congress, 219.
Professional Advertising, 137.
Resignation of Dr. Head, 218.
Sunshine and Shadow, 544.
The Congress Programme—Important Corrections, 388.
The Congress Report, 812.
The Dental Protective Association, 467.
The Ethical War in Minneapolis, 467.
The Importance of attending the Columbian Dental Congress, 384.
The Last Days preceding the Congress, 547.
The Pacific Coast Dentist, 627.
The Passing Year, 932.

Editorial:
The Present and Future of Scientific Publications, 464.
The Three Conventions, 303.
The World's Columbian Dental Congress, 809.
To Contributors and Correspondents, 305.
To Correspondents, 812.
To Subscribers, 140.
To Whom belongs the Honor of Discovery, 930.
Whither are we drifting? 215.
World's Columbian Dental Congress—New Order of Business, 218.
Editorial Correspondence: World's Columbian Dental Congress, 698.
Educating the Public, 632.
Education for its own sake, 552.
Effect of Imagination, 936.
Effect upon the Tissues of the Oral Cavity produced by the Administration of Certain Drugs, 270.
Elements of Chemistry and Dental Materia Medica, Review of, 468.
Enamel, A Contribution to the Study of the Development of, 881.
Congenital Defects of, 759.
Surfaces for Gold Crowns, 40.
English Tube Teeth and their Uses, 742.
Entertainment of the Medical Profession at the World's Fair, 640.
Erosion, A Study of, 241.
of the Teeth, 249.
Ether-Inhaler, Use of a New, 401.
Ethical War in Minneapolis, 467.
Ethics, Professional, 705.
Ethyl Chloride, 174.
Eucalyptus Oil, Alleged Death from, 880.
Europhen, 176.
Evans, Dr. George. Enamelling the Surfaces of Gold Crowns, 40.
Crown- and Bridge-Work, Review of, 390.
Examining Boards, The Power of, 10.
Pennsylvania State, 480.
Expenses to the Congress, 627.
Experiences in treating Cleft Palates, 406.
Experiments with Bichloride of Mercury, 753.
Exposed Pulps, The Treatment of, 499.
Extracting, 311.
as a Conservative Treatment, 18.

F.

Facts showing the Relationship of the Dental Interarticulation, with more or less Obscure Pains about the Mouth and Jaws, 788.
Failure in Dental Operations, 25.
Faught, L. Ashley, D.D.S. Causes of Failure in Dental Operations, 25.
What are the Best Materials to enter into the Composition of Temporary Fillings? 419.
Fillebrown, Thomas, M.D., D.M.D., on A New Apparatus for maintaining Anæsthesia, 757.
The Uses of Hypnotism in Dentistry, 355.
Filling-Material, Amalgam as a, 572.
Materials, A Few Thoughts on the Combination of, 570.
Roots with Gutta-Percha dissolved in Chloroform, 267.
Fisher, Dr. Harvey, Resolutions on the Death of, 703.
Fletcher, M. H., R.P., Pan-American Medical Congress, 226.
Formulaire Pratique pour les Maladies de la Bouche et des Dents. Par G. Viau. Review of, 628.
Freeman, S., D.D.S., on The Use of Gutta-Percha as a Root-Canal Filling, 662.
Friedrichs, Dr., on Nitrate of Silver, 238.
Further Consideration of the Subject of Dentition, 27.
Use of Nitrate of Silver, 707.

G.

Genese, David, D.D.S., on Crowns, 413.
Georgia State Dental Society, 639.
Girdwood, John, L.D.S., D.D.S., on English Tube Teeth and their Use, 742.
Gold and Amalgam as a Base for Filling, 170.
Watt's Crystal, 359.
Granting Certificates to Undergraduates, 10.
Guilford, S. H., D.D.S. Orthodontia (New Edition), 389.
on The Teeth and Hair: their Homology and Pathological Intimacy, 826.
Gutta-Percha and Chloroform as a Root-Filling, 267.

H.

Harlan, Dr., on Europhen, Trichloracetic Acid, and Syrup of Iron Chloride, 177.
Harris, Dr. E. M., Resolutions of Respect to, 469.
Harvard Odontological Society, 319, 446, 613, 672.
Herbst Method of treating Pulps, 310.
High-Tension Circuits for Dental Work, The Disadvantages of, 483.
Histology, Pathology, and Bacteriology, Review of, 146.
Homology and Pathological Intimacy of the Teeth and Hair, 826.
Hopkins, Frederick F., on Watt's Crystal Gold, 359.
Howe, Dr. J. Morgan. A Contribution to the Study of Erosion, 241.
Hugenschmidt, A. C., M.D., D.D.S., on A Case of Fracture and Suppuration around the Incisive Alveolar Portion of the Superior Maxillary Bone, and Reimplantation in the same of a Lateral Incisor Eight Days after the Injury, 842.

Hughes, Dr. C. H., on Pan-American Medical Congress, 232.
Hunt, Dr., on Materia Medica and Therapeutics, 173.
Hydrogen Peroxide Solutions, Some of the Properties of, 646.
Hydronaphthol in Treatment of Cholera, 79.
Hyperostosis, 852.
Hypertrophy (Hyperplasia), 8.
Hypnotism in Dentistry, The Uses of, 355.
in Medicine, The Uses of, 337,

I.

Illinois State Dental Society, 399, 639.
Implantation, Comments on, 476.
Importance of attending the Columbian Dental Congress, 384.
Incidents of Practice, 282, 371.
Increase of Leucocytes in the Blood after Cold Baths, 876.
Increased Vitality hinders Bacteria in Caries, 879.
Indications justifying the Extraction of Teeth, 312.
Inflammation, Terminations of, 8.
The Doctrine of, 1.
Injuries and Diseases of the Mouth and their Treatment, 321.
Internal Administration of Certain Drugs, The Effect upon the Tissues of the Oral Cavity produced by, 270.
International Congress of Medicine, The Eleventh, 313.
Dental Publishing Company, Annual Meeting of, 228.
Medical Congress, Rome, Italy, Reduction in Fares to, 638.
Investigations Relative to Prehistoric Crania, 278.
Iodine, Carbolic Acid, and Chloral in Skin-Diseases, 160.
Iodoform *versus* Aristol, 877.
Iron, Syrup of Chloride of, 182.
Ives, Dr. C. F. The Treatment of Nearly-Exposed Pulps, 499.

J.

Jack, Louis, D.D.S., on The Power of Examining Boards, 10.

K.

Kelley, H. A., D.M.D. A Study of the Diseases of the Peridental Membrane, 102.
Kingsley, Dr. Charles W., Obituary, 935.
Kirk, E. C., D.D.S. Crystal Mat Gold, 97.
Lime Formations in the Pulp-Chamber, 894.

L.

Ladd, Babson S., on Some of the Rights and Duties of Dentists at Common Law, 586.
Last Days preceding the Congress, 547.
Latham, Vida A., D.D.S., F.R.M.S., on Palatal Diseases as applied to Dentistry, 764.
Laurence, Dr. Ambrose, Obituary, 470.
Lecaudey, Dr. E., on Treatment of Abscess of the Maxillary Sinus, 800.
Letters from a Mother to a Mother, Review of, 628.
Leucocytes in the Blood after Cold Baths, Increase of, 876.
Levy, Dr. Frederick A., Obituary, 396.
New Jersey State Dental Society, Resolutions of Respect to, 470.
Lime Formations in the Pulp-Chamber, 894.
Living in Chicago during the Fair, 629.
Local Anæsthetic, A New Coca-Base as a, 237.
Lord, Dr. Benjamin. A Brief Review, 15.
A Few Thoughts on the Combination of Filling-Materials, 570.
Lower Vulcanite Dentures, A Simple and Efficient Strengthener for, 876.

M.

McManus, Dr. James, Notes on the History of Anæsthesia, 900.
Manning, Frederick A., M.D. A Manual of Physiology, 223.
Manual Training, 107, 139.
Massachusetts Dental Society, 400.
Materia Medica and Therapeutics, Review of, 146.
Matrices, 55.
Mayer, Dr., on Does the Quality of Amalgam depend on Atomic Formulæ? 676.
Mechanics in Dentistry, 312.
Medical Congress, Pan-American, 219, 229, 232, 634.
Profession at the World's Fair, Entertainment of, 640.
Memorial of the British Dental Association to the General Medical Council of Great Britain, 633.
Method of inducing Local Anæsthesia by Cocaine, 775.
Methods advocated for obviating the Necessity of extracting Devitalized Tooth-Pulps, 731.
Methods of Filling Teeth, Review of, 141.
Miller, Dr. W. D., on Methods advocated for obviating the Necessity of extracting Devitalized Tooth-Pulps, 731.
Mills, Dr. G. A., on Riggs's Disease: What is it, and can it be cured? 655.
Morrison, Dr., on Transplantation, 55.
Mouth, Injuries and Diseases of, and their Treatment, 321.
"Mrs. M. W. J." Letters from a Mother to a Mother, Review of, 628.

N.

National Association of Dental Faculties, 560, 707.
Board of Dental Examiners, 636, 712.
Native Hawaiians, Crania of, 871.
New Jersey State Dental Society, 560.
New York College of Dentistry, Alumni, 235.
New York Odontological Society, 31, 281, 371, 421, 501, 588, 676.
Nitrate of Silver, 398.
Dr. Friedrichs on, 238.
Dr. Hunt on, 175.
Dr. Peirce on, 153.
Dr. Stockton on, 707.
Nitrous Oxide Gas Mortality, 398.
Nixon, Dr. Franklin M, Obituary, 550.
Nomenclature as applied to Dentistry, 863.
Northern Ohio Dental Association, 236.
Note-Book for Dental Students (Dental Anatomy and Physiology), Review of, 222.
Notes and Comments, 310, 398, 475, 552, 630, 706, 813, 869, 937.
Notes on the History of Anæsthesia, 900.

O.

Obituary: Allport, Dr. W. W., 391, 469, 629.
Beale, Dr. Alonzo, 146.
Fisher, Dr. Henry, 703.
Harris, Dr. E. N., 469.
Kingsley, Dr. Charles W., 935.
Laurence, Dr. Ambrose, 470.
Levy, Dr. Frederick A., 396, 470.
Nixon, Dr. Franklin M., 550.
Park, Dr. Edgar, 224.
Register, Dr. John E., 78.
Rehfuss, Dr. William F., 395.
Roberts, Dr. George, 147.
Wardlaw, Dr. W. C., 867.
Odontographic Society of Chicago, 235.
Odontological Society of New York, 31, 281, 421, 501.
of Pennsylvania, 60, 126, 294, 440, 529, 607, 855, 917.
Ohio State Dental Society, 237.
Operative Dentistry, 54.
Oral Hygiene, 399.
Origin of the Practical Application of Anæsthesia in Surgery, 942.
Orthodontia, A Review of, 389.
Its Present Status. What are the Simplest and Most Universally Applied Forms of Apparatus, and Most Efficient Retaining Fixtures? 844.
Some Principles governing the Development of Facial Contours in the Practice of, 776.
Osgood Hamilton, M.D., on The Uses of Hypnotism in Medicine, 337.
Our Duty in Regard to the World's Columbian Dental Congress, 471.
Oxide of Copper Cement, 38.
Oxychinaseptol, or Diaphtherin, 239.

P.

Page, Edward, M.D., D.M.D., on Amalgam as a Filling-Material, 572.
Reply to Dr. Taft, 868.
Palatal Diseases as applied to Dentistry, 764.
Palmer, S. B., M.D.S., on Manual Training, 107.
on The Use of Clasps—Bonwill Method, 353.
Pan-American Medical Congress, 219, 229, 232, 634.
New By-Laws of, 636.
Park, Edgar, D.D.S, Obituary, 224.
Patrick, Dr. J. J. R., on Investigations Relative to Prehistoric Crania, 278.
Patterson, Dr. J. D., on The Effect upon the Tissues of the Oral Cavity produced by the Internal Administration of Certain Drugs, 270.
Peirce, C. N., D.D.S. Affections of the Teeth, 641.
Dental Diagnosis, 889.
Further Considerations of the Subject of Dentition, 27.
Nitrate of Silver, 153.
Nixon, Dr. Franklin M., Obituary, 550.
Pennsylvania Odontological Society, 60, 126, 294, 440, 529, 607, 855, 917.
State Dental Society, 566, 622.
State Examining Board, 480.
Pental, 173.
Peridental Membrane, A Study of the Diseases of, 102.
Perry, Safford G., D.D.S. Erosion of the Teeth, 249.
Phenocoll Hydrochloride, 946.
Philadelphia County Dental Society, Officers of, 159.
Physiology, A Manual of, 223.
Plastic Filling-Material, 860.
Plastics, 59.
Post-Graduate Instruction, The Value of, 486.
Prehistoric Crania from Central America, 914.
Present and Future of Scientific Publications, 464.
Principles governing the Development of Facial Contour in the Practice of Orthodontia, 776.
Professional Ethics, 705.
Prudence and Gutta-Percha in Crown- and Bridge-Work, 782.
Pulp-Canals, Some Changes that take place in and around, 794.
Pulp-Chamber, Line Formations in the, 894.
Pulps, The Herbst Method of treating, 310.
Putrescent Pulp, Treatment of, 168.
Pyorrhœa Alveolaris, 630.
Important Announcement, 938.
The Treatment of, 490.

Q.

Questions and Answers for Dental Students, Review of, 76.

R.

Recent Patents, 79, 159, 477, 637, 817, 874, 940.
Reduction of Fares to International Medical Congress, Rome, Italy, 638.
Regulating Teeth, Twisted Wire for, 247.
Regulation and Retention of the Teeth—Angle's System, 223.
Rehfuss, Dr. Wm. F., Obituary, 395.
Reply to Dr. Taft, 868.
to Dr. Page, 704.
Report of the Committee on Dental Nomenclature, 837.
Researches in Bacteriology, 944.
Resolution, 8.
Rich, Dr. John B. Report on the Census Law, 362.
Richmond, Dr. C. M. Crown- and Bridge-Work, 81, 161, 481.
Riggs's Disease—What is it, and can it be cured? 655.
Reilly, James A., D.M.D. Unnecessary Pain in Dental Operations, 164.
Rise, Fall, and Revival of Dental Prosthesis, Review of, 221.
Root-Canals, Treatment of Infected, with Kalium and Natrium, 791.
Filling, Gutta-Percha and Chloroform as a, 267.
Roy, F. A. Professional Ethics, 705.
Rubber, Weighted, 311.
Rymer, James F. Note-Book for Dental Students (Anatomy and Physiology), 222.

S.

Sage, Frank W., D.D.S., Effect of Imagination, 936.
Schreier, Dr. Emil, on The Treatment of Infected Root-Canals with Kalium and Natrium, 791.
Shepherd, James, D.M.D., on Tact, 262.
Silver Nitrate, 153, 175, 238, 398, 707.
Simonton, Dr. W. F., Gold and Amalgam as a Base for Filling, 170.
Simonton, Dr. W. S. To make Joints Tight with Amalgam, 582.
Smith, P. T., D.D.S., on Cell Metamorphosis, 659.
Society Meetings, Reports of: American Academy of Dental Science, 118, 433, 519, 602, 662, 801, 844.
American Dental Association, 34, 109, 173, 362, 477.
California State Dental Society, 480.
Central Dental Association of Northern New Jersey, 66, 209, 535.
Chicago Dental Club, 452.
Colorado State Dental Association, 623.
Connecticut Valley Dental Society, 719.
Society Meetings:
Georgia State Dental Society, 639.
Harvard Odontological Society, 317, 446, 672.
Illinois State Dental Society and Iowa State Dental Society—Joint Meeting, 399, 639.
Massachusetts Dental Society, 400.
Missouri State Dental Association, 480.
New Jersey State Dental Society, 560.
New York Odontological Society, 31, 281, 371, 421, 501, 588, 676.
North Carolina State Dental Society, 319.
Northern Ohio Dental Association, 236.
Odontographic Society of Chicago, 235.
Odontological Society of Pennsylvania, 60, 126, 294, 440, 529, 607, 855, 917.
Ohio State Dental Society, 237.
Pennsylvania State Dental Society, 560, 622.
St. Louis Dental Society, 236.
San Francisco Dental Association, 159.
South Carolina State Dental Association, 560, 636.
Southern Dental Association, 639, 815.
The Dental Society of the State of New York, 400.
Woman's Dental Association of the United States, 875, 926.
Some of the Causes of Failure in Dental Operations, 21.
Some of the Properties of Hydrogen Peroxide Solutions, 646.
Some of the Rights and Duties of Dentists at Common Law, 586.
South Carolina State Dental Association, 560, 636.
Southern Dental Association, 639, 815.
Special Notice—Associations at Chicago, 639.
Stainton, C. W., D.D.S., on Crownless Teeth, 196.
Stewart, Carrie M., on Experiments with Bichloride of Mercury, 753.
St. Louis Dental Society, 236.
Stockwell C. T. Further Use of Nitrate of Silver, 707.
Study of the Diseases of the Peridental Membrane, 102.
Sunshine and Shadow, 544.
Suppuration, 8.
Swift, Dr. Arthur L., on The Treatment of Putrescent Pulps, 168.
Syrup of Iron Chloride, 182.

T.

Tact, 262.
Taft, Charles H. Reply to Dr. Page, 704.
Talbot, Eugene S., M.D., D.D.S. A Study of the Degeneracy of the Jaws of the Human Race, 219.
Henry P., Ph.D. Some of the Properties of Hydrogen Peroxide Solutions, 646.

Teeth, Affections of the, 641.
and Hair: Their Homology and Pathological Intimacy, 826.
Crownless, 196.
Erosion of, 249.
Indications justifying the Extraction of, 312.
of the Lower Jaw at Birth, 721.
Regulating, with Twisted Wire, 247.
Temporary Fillings, What are the Best Materials for? etc., 419.
Thayer, W. Irving, M.D. An Abnormal Position of the Left Central, 653.
The Clumsy Dentist, 476.
"The Cobalt of Herbst," 476.
The Congress Report, 812.
The Dentist as a Prescriber of Drugs, 567.
The Forms of Edentulous Jaws in the Human Subject, 872.
The Functions of the Schools, 631.
The Genese Crown, 399.
The Hydrogen Dioxide Controversy, 943.
The Pacific Coast Dentist, 627.
The Passing Year, 932.
The Pedigree of the Central Incisor, 833.
The Teeth and Hair: Their Homology and Pathological Intimacy, 826.
The Test of Strength, 937.
The Three Conventions, 303.
The Use of Gutta-Percha as a Root-Canal Filling, 662.
Therapeutic Use of Syrup of Chloride of Iron, 552.
Thompson, A. H., D.D.S., on The Pedigree of the Central Incisor, 833.
To Contributors and Correspondents, 305.
To Correspondents, 812.
To Deodorize Iodoform, Creosote, and Guaiacol, 945.
To make Joints Tight with Amalgam, 582.
To Whom belongs the Honor of Discovery? 930.
Transplantation, 55.
Treatment of Abscess of the Maxillary Sinus, 800.
of Infected Root-Canals with Kalium and Natrium, 791.
of Nearly Exposed Pulps, 499.
of Putrescent Pulps, 168.
of Pyorrhœa Alveolaris, 490.
Trichloracetic Acid, 182.
Truman, James, D.D.S. Treatment of Pyorrhœa Alveolaris, 490.
Twisted Wire for regulating Teeth, 247.

U.

Unnecessary Pain in Dental Operations, 164.
Use of Clasps—Bonwill Method, 353.
of Hypnotism in Dentistry, 355.
in Medicine, 337.

V.

Value of Post-Graduate Instruction, 486.
Van Orden, L., M.D., on Some Facts showing the Relationship of the Dental Interarticulation, with more or less Obscure Pains about the Mouth and Jaws, 788.
Vegetable Mercury of Brazil, 878.

W.

Wardlaw, W. C., D.D.S., Obituary, 867.
Warren, George W., D.D.S. A Compend of Dental Pathology and Dental Medicine (Second Edition), 549.
New Edition of Richardson's Mechanical Dentistry, 934.
Watt, Dr. George. Obituary, 307.
Dr. J. E. Anæsthesia, and the Use of a New Ether-Inhaler, 401.
Watt's Crystal Gold, 359.
Weighted Rubber, 311.
What are the Best Materials for Temporary Fillings? etc., 419.
Whither are we drifting? 215.
Whitney, J. M., D.D.S., on Among the Ancient Hawaiians, 735.
Woman's Dental Association of the United States, 875, 926.
World's Columbian Dental Congress, 154, 218, 229, 553, 698, 809.
Important Notice, 479.
Our Duty in Regard to, 471.
Special Notice, 478.
Wyoming, An Act to Regulate Dentistry in, 720.

Z.

Zsigmondy, Dr. Otto, on Congenital Defects of the Enamel, 759.

### International Dental Journal.

### PROSPECTUS FOR THE YEAR 1893.

The coming year will be the fifth of this journal in its present form, under the auspices of the International Dental Publication Company. It is with a sense of pride we look upon the character of the articles which have appeared in its numbers during this period. Those who have the complete files will find a large amount of solid, serviceable matter in both scientific and practical subjects.

For the next year this journal shall remain the vehicle for the publication of the proceedings of the **Odontological Society of New York**, the **American Academy of Dental Science of Boston, Harvard Odontological Society of Boston,** and the **Odontological Society of Pennsylvania.** It will also contain a full report of the proceedings and abstracts from the papers presented to the **American Dental Association.** The same attention will be given to the papers of the **World's Columbian Dental Congress,** so far as they may become available.

The papers upon **Crown- and Bridge-Work by Dr. Richmond** will be continued. These furnish a description of methods with results more striking, in this specialty, for their exactness and practicability than those of many others who have been devoted to this line of work.

This journal extends its fellowship to all other journals devoted to the good of dentistry, and has no antagonism towards any. The field of dentistry is too large to be covered by a single journal, and those who would keep abreast must have access to all the leading dental periodicals.

We shall, however, endeavor in the future to embody in our pages reviews and abstracts of good papers from other journals.

We ask of the dental profession their fullest support, that we may be enabled to carry out the ends we have in view in a liberal manner. Upon the extent of this support depends the freedom with which this may be done. We point with satisfaction to our previous work as an indication of what will be effected in the near future.

With an expression of gratefulness for the speedily increasing patronage, we look forward with reasonable hope to enlarged usefulness in the coming year.

The INTERNATIONAL DENTAL PUBLICATION CO.,

716 Filbert Street, PHILADELPHIA, PA.

### ORDER FOR SUBSCRIPTION.

.......................................................... 189

M..........................................................................

..........................................................................

Enclosed find $2.50, for which please send the

**INTERNATIONAL DENTAL JOURNAL**

for one year, beginning with the January number, 1893, to

*Name*, ..........................................................................

*Street and No.*, ..........................................................................

*City or Town*, ..........................................................................

*County*, ..........................................................................

*State*, ..........................................................................

---

Remove this slip from the book, fill it out carefully, and enclose it, together with $2.50, to any one of the agents named on our list.

### IMPORTANT NOTICE.

For the convenience of subscribers to the INTERNATIONAL DENTAL JOURNAL, who often find it difficult to remit small amounts through the mail, we have established agencies in different localities (in most cases with dealers in Dental Goods, with whom dentists have frequent business transactions), WHERE EITHER NEW OR RENEWAL OF SUBSCRIPTIONS MAY BE PAID.

### International Dental Publication Co.,

PUBLISHERS.

### LIST OF AGENTS.

| NAME. | ADDRESS. | BUSINESS. |
|---|---|---|
| Dr. Walker G. Browne | Atlanta, Ga | Dental Supplies. |
| Fred. W. Smith | Binghamton, N. Y | Dental Depot. |
| F. M. Allen, D.D.S | Birmingham, Ala | Dental Depot and Dentist. |
| Buffalo Dental Mfg. Co | 587 and 589 Main Street, Buffalo, N. Y | Dental Depot. |
| Boston Dental Mfg. Co | 174 Tremont Street, Boston | Dental Supplies. |
| B. L. Knapp & Co | 161 Tremont St., Boston, Mass | Dentists' Materials. |
| Illinois Dental Mfg. Co | 85 Fifth Ave., Chicago, Ill | Dental Supplies. |
| S. A. Crocker & Co | 119 W. Fifth St., Cincinnati, Ohio | Ohio Dental and Surg. Depot. |
| George W. Fels | 139 W. Sixth Street, Cincinnati, Ohio | Dental Depot. |
| M. A. Spencer & Co. | 134 W. Seventh Street, Cincinnati, O. | Surg. and Dent. Instruments. |
| J. Durbin | Denver, Col | Dental Goods. |
| Marshall Dental Mfg. Co | 309 Seventh Street, Des Moines, Iowa. | |
| Keller Dental Co | Fort Wayne, Ind. | Dental Manufacturers. |
| C. B. Woodward & Co | Fort Wayne, Ind. | Dental Depot. |
| E. S. Holmes | Grand Rapids, Mich | Dentist. |
| W. C. Messinger | Hartford, Conn | Dental Supplies. |
| J. E. Bodine & Co | Indianapolis, Ind. | Dental Depot. |
| Dr. A. W. Clark | Jefferson, N. Y | Dental Supplies. |
| R. I. Pearson & Co | 906 Grand Avenue, Kansas City, Mo | Surgical and Dental Depot. |
| T. P. Wagoner | 64 Main Street, Knightstown, Ind | Dental Goods. |
| L. S. Keener | Knoxville, Tenn | East Tennessee Dental Depot. |

| NAME. | ADDRESS. | BUSINESS. |
|---|---|---|
| Wells Dental Depot | Corner Spring and First Streets, Los Angeles, Cal. | |
| L. J. Frazee | 438 W. Walnut Street, Louisville, Ky. | Louisville Dental Depot. |
| E. G. Fowler | Montgomery, Ala. | Dental Depot. |
| Morrison Bros | Nashville, Tenn | Dental Depot. |
| Osman & Cook | Newark, N. J., Box 265 | Newark Dental Depot. |
| E. L. Washburn & Co. | New Haven, Conn. | Dental Depot. |
| Stuart & Adams | New Orleans, La. | Dental Depot. |
| C. Ash & Sons | 30 East 14th St., New York | Dental Goods. |
| E. Steiger & Co. | 32 Park Place, New York. | |
| Dr. C. E. Fitzgerald | Paris, Tenn | Dentist. |
| L. D. Caulk | 1219 Arch Street, Philadelphia, Pa. | Manufacturer of Caulk's Filling Materials. |
| J. B. Dunlevy | Pittsburgh, Pa. | Dental Depot. |
| Lee S. Smith | 52 6th St., Pittsburgh, Pa. | Dental Depot. |
| George C. Frye | Portland, Maine | Dental Depot. |
| Dr. F. W. Seabury | 294 Westminster Street, Providence, R. I. | Dentist. |
| Charles Oehlmann | Quincy, Ill. | Dental Depot. |
| A. Grant | 917 Main St., Richmond, Va. | Dental Depot. |
| James W. Edwards, | 5½ Kearny Street, San Francisco, Cal. | |
| J. H. A. Folkers & Bro. | 815 Market Street, San Francisco, Cal. | |
| Wm. M. Williams | Springfield, Mass | Dental Depot. |
| John Rowan | St. Louis, Mo. | Dental Depot. |
| St. Louis Dental Mfg. Co. | St. Louis, Mo. | Dental Depot. |
| Minnesota Dental Mfg. Co. | St. Paul, Minn. | |
| M. F. Patterson | St. Paul, Minn. | Dental Goods. |
| H. C. Leyden | Syracuse, N. Y. | Dental Depot. |
| The Buckhart Dental Supply Co. | 930 Pacific Avenue, Tacoma, Wash | Dental Goods. |
| J. P. Stansell | Temple, Texas | Dental Depot. |
| Ransom & Randolph | Toledo, Ohio | Dental Depot. |
| S. B. Chandler Dent. Mfg. Co. | Toronto, Canada | Dental Goods. |
| C. H. Hubbard | 44 Adelaide Street W., Toronto, Canada. | |
| E. J. Lewis | 1215 Pennsylvania Ave., Washington, D.C. | Dealer in Dental Materials. |
| Robinson & Armstrong | Watertown, N. Y. | Dental Supplies. |

### FOREIGN AGENTS.

| NAME. | ADDRESS. | BUSINESS. |
|---|---|---|
| C. Ash & Sons | Broad St., Golden Square, London, England | Dental Goods. |
| Dental Mfg. Co., Limited | 6 Lexington Street, West London, England | Dental Goods. |
| P. A. Kolliker & Co. | Bahnhofstrasse 64, Zurich, Switzerland | Dental Depot. |
| C. A. Lorenz | Königsplatz 1, Leipsig, Germany | Dental Goods. |

### Announcement

### ··for the Year 1894··

This number closes the fourteenth volume of this Journal, and the sixth under its present management.

The duty of supplying the dental profession with a dignified and liberal vehicle for the conveyance of information upon every topic relative to dentistry, has been steadily kept in view. It has furnished, through the proceedings of the various Dental Societies, which have used it as their publishing medium, articles upon a multitude of scientific subjects, and the discussions of these organizations have been full of practical suggestions and of incalculable value. We attach much importance to matter coming fresh from the minds of those in actual practice, conveyed in their own words.

In the editorial department the aim has been to be fearless and independent in the expression of opinions upon matters of polity, as affecting the status of dentistry.

Its advertising pages have conveyed information of the means of procuring appliances and materials from sources which, without this channel, would have lacked the same publicity.

The record we have made in the past will be our guide in the future, satisfied that the support we have received will be continued. We are conscious that the INTERNATIONAL DENTAL JOURNAL is patronized by the leading practitioners in all sections, and while recognizing this encouraging fact, we have reason to feel that the Journal has a claim upon every dentist, not only to renew promptly his own subscription, but to aid in extending its influence among the large body of dentists who have failed to appreciate its earnest work in the interests of the profession.

THE INTERNATIONAL DENTAL JOURNAL,

716 Filbert Street, Philadelphia.

SUBSCRIBE FOR THE

### International Dental Journal,

EDITED BY JAMES TRUMAN, D.D.S.

### Published by the International Dental Publication Company.

LOUIS JACK, PRESIDENT, Philadelphia.
BENJAMIN LORD, VICE-PRESIDENT, New York.

C. N. PEIRCE, TREASURER, Philadelphia.
GEO. S. ALLEN, SECRETARY, 51 West Thirty-seventh St., New York.

**Publication Office, 716 Filbert St., Philadelphia, Pa.**

### STOCKHOLDERS.

| | |
|---|---|
| Geo. S. Allan | New York, N.Y. |
| R. R. Andrews | Cambridge, Mass. |
| H. A. Baker | Boston, Mass. |
| A. E. Baldwin | Chicago, Ill. |
| W. C. Barrett | Buffalo, N.Y. |
| S. T. Beale, Jr. | Philadelphia, Pa. |
| C. S. Beck | Wilkesbarre, Pa. |
| G. V. Black | Jacksonville, Ill. |
| C. F. W. Bodecker | New York, N.Y. |
| E. A. Bogue | New York, N.Y. |
| W. G. A. Bonwill | Philadelphia, Pa. |
| C. A. Brackett | Newport, R.I. |
| Thomas Bradley | New York, N.Y. |
| Edward C. Briggs | Boston, Mass. |
| Albert H. Brockway | Brooklyn, N.Y. |
| A. E. Brown | Chicago, Ill. |
| Wm. Carr | New York, N.Y. |
| Geo. H. Chance | Portland, Oregon. |
| G. F. Cheney | St. Johnsbury, Vt. |
| Dwight M. Clapp | Boston, Mass. |
| John T. Codman | Boston, Mass. |
| Chas. D. Cook | Brooklyn, N.Y. |
| J. N. Crouse | Chicago, Ill. |
| Edw. T. Darby | Philadelphia, Pa. |
| H. S. Draper | Boston, Mass. |
| Wm. H. Dwinelle | New York, N.Y. |
| A. R. Eaton | Elizabeth, N.J. |
| Albert T. Emery | Boston, Mass. |
| Chas. J. Essig | Philadelphia, Pa. |
| J. N. Farrer | New York, N.Y. |
| T. Fillebrown | Portland, Me. |
| W. B. Finney | Baltimore, Md. |
| T. A. Fletcher | New York, N.Y. |
| M. W. Foster | Baltimore, Md. |
| Chas. E. Francis | New York, N.Y. |
| E. C. Fundenberg | Pittsburg, Pa. |
| W. F. Fundenberg | Pittsburg, Pa. |
| W. H. Fundenberg | Pittsburg, Pa. |
| E. S. Gaylord | New Haven, Conn. |
| J. P. Geran | Brooklyn, N.Y. |
| A. H. Gilson | Boston, Mass. |
| S. H. Guilford | Philadelphia, Pa. |
| John I. Hart | New York, N.Y. |
| O. E. Hill | Brooklyn, N.Y. |
| J. F. P. Hodson | New York, N.Y. |
| J. Morgan Howe | New York, N.Y. |
| Robert Huey | Philadelphia, Pa. |
| A. O. Hunt | Iowa City, Iowa. |
| Louis Jack | Philadelphia, Pa. |
| V. H. Jackson | New York, N.Y. |
| C. R. Jefferis | Wilmington, Del. |
| W. T. La Roche | Harrington Park, N.J. |
| W. K. Leech | Philadelphia, Pa. |
| Wilbur F. Litch | Philadelphia, Pa. |
| J. Bond Littig | New York, N.Y. |
| Benjamin Lord | New York, N.Y. |
| Geo. W. Lovejoy | Montreal, Ont. |
| John S. Marshall | Chicago, Ill. |
| H. J. McKellops | St. Louis, Mo. |
| Charles McManus | Hartford, Conn. |
| James McManus | Hartford, Conn. |
| Dan'l Neal McQuillan | Philadelphia, Pa. |
| Frederic A. Merrill | Boston, Mass. |
| Horatio C. Meriam | Salem, Mass. |
| W. D. Miller | Berlin, Germany. |
| Geo. T. Moffatt | Boston, Mass. |
| W. E. Page | Boston, Mass. |
| S. B. Palmer | Syracuse, N.Y. |
| C. B. Parker | Brooklyn, N.Y. |
| D. D. Peabody | Stoneham, Mass. |
| C. N. Peirce | Philadelphia, Pa. |
| S. G. Perry | New York, N.Y. |
| W. A. Phreaner | Philadelphia, Pa. |
| Chas. E. Pike | Philadelphia, Pa. |
| W. E. Pinkham | New York, N.Y. |
| W. H. Potter | Boston, Mass. |
| Chas. P. Pruyn | Chicago, Ill. |
| H. C. Register | Philadelphia, Pa. |
| Z. T. Sailer | New York, N.Y. |
| L. D. Shepard | Boston, Mass. |
| Geo. R. Shidle | Pittsburg, Pa. |
| Eugene H. Smith | Boston, Mass. |
| B. Holly Smith | Baltimore, Md. |
| H. A. Smith | Cincinnati, Ohio. |
| S. G. Stevens | Boston, Mass. |
| W. X. Sudduth | Minneapolis, Minn. |
| Eugene S. Talbott | Chicago, Ill. |
| Ambler Tees | Philadelphia, Pa. |
| J. D. Thomas | Philadelphia, Pa. |
| James Truman | Philadelphia, Pa. |
| Wm. H. Trueman | Philadelphia, Pa. |
| W. W. Walker | New York, N.Y. |
| I. F. Wardwell | Stanford, Conn. |
| G. W. Warren | Philadelphia, Pa. |
| T. S. Waters | Baltimore, Md. |
| Geo. W. Weld | New York, N.Y. |
| Julius G. W. Werner | Boston, Mass. |
| Jacob L. Williams | Boston, Mass. |
| R. B. Winder | Baltimore, Md. |
| C. A. Woodward | New York, N.Y. |
| J. A. Woodward | Philadelphia, Pa. |
| W. A. Woodward | New York, N.Y. |
| W. J. Younger | San Francisco, Cal. |

The officers of the INTERNATIONAL DENTAL PUBLICATION COMPANY serve without salary, the investments of the stockholders merely guarantee the success of the enterprise, ALL PROFITS being devoted to the enlargement or improvement of the INTERNATIONAL DENTAL JOURNAL.

**The support of every dentist having the welfare of his profession at heart is earnestly solicited. Subscription, $2.50 per Annum.**

*THE INTERNATIONAL DENTAL PUBLICATION CO.*

